# Supplementary material for: Dissipation Kinetics and Safety Evaluation of Flonicamid in Four Various Types of Crops
Source: Molecules. 2022 Dec 6;27(23):8615. doi: 10.3390/molecules27238615 (PMC9738400; doi:10.3390/molecules27238615)
Supplement: Supplementary file 1 [file molecules-27-08615-s001.zip › molecules-2013549-supplementary.pdf]

Table S1. Purification combination and dosage of PSA and $MgSO_4$ in 4 typical matrices

Sample	Weight (g)	NaCl+$MgSO_4$(g)	PSA+ $MgSO_4$(mg)
Peach	10	3 + 2	100 + 100<br>150 + 50<br>50 + 150
Cucumber	10	3 + 2	100 + 100<br>150 + 50<br>50 + 150
Cabbage	10	1 + 4	100 + 100<br>150 + 50<br>50 + 150
Cottonseed	5	1 + 4	100 + 100<br>150 + 50<br>50 + 150

Table S2. Gradient elution program

Time （min）	Flow rate （mL/min）	Mobile phase（%）	
		0.05% Formic acid water	Methanol
0.00	0.30	90	10
2.50	0.30	10	90
3.50	0.30	10	90
3.51	0.30	90	10
5.00	0.30	90	10

Table s3. Field test sites, crop varieties and test types

Crop types	Test address	Abbreviation of test sites	Crop varieties	Test types
Peach	Xincheng District, Hohhot City, Inner Mongolia Province	Inner Mongolia	Youpan	Terminal residue
	Xincheng District, Hohhot City, Inner Mongolia Province	Inner Mongolia	Youpan	Terminal residue
	Yuci District, Jinzhong City, Shanxi Province	Shanxi	Okubo	Terminal residue
	Sujiatuo Town, Haidian District, Beijing Province	Beijing	Xinchuanzhongdao	Residual dissipation and terminal residue
	Daiyue District, Tai'an City, Shandong Province	Shandong	Kangfang	Residual dissipation and terminal residue
	Gongyi City, Zhengzhou City, Henan Province	Henan	Zhongyou5	Residual dissipation and terminal residue
	Changshu City, Suzhou City, Jiangsu Province	Jiangsu	Jingchun	Terminal residue
	Huanghua Town, Changsha City, Hunan Province	Hunan	Cuimi	Residual dissipation and terminal residue
	Huaxi Town, Guiyang City, Guizhou Province	Guizhou	Cuimi	Terminal residue
Cucumber	Taizihe District, Liaoyang City, Liaoning Province	Liaoning	Lvrou	Residual dissipation and terminal residue
	Saihan District, Hohhot City, Inner Mongolia Province	Inner Mongolia	Shuika	Terminal residue
	Weiyang District, Xian City, Shanxi Province	Shanxi	Dinghao	Terminal residue
	Panggezhuang Town, Daxing District, Beijing	Beijing	Zhongnong26	Residual dissipation and terminal residue
	Taishan District, Tai'An City, Shandong Province	Shandong	Mingxing1	Residual dissipation and terminal residue
	Gongyi City, Zhengzhou City, Henan Province	Henan	Jinyou1	Terminal residue
	Laixi City, Qingdao City, Shandong Province	Shandong	Xiafeng1	Terminal residue
	Chaohu City, Hefei City, Anhui Province	Anhui	Jingyan4	Terminal residue
	Xinbang Town, Songjiang District, Shanghai Province	Shanghai	Heiliangwang	Terminal residue
	Chunhua Town, Changsha City, Hunan Province	Hunan	Xiangjin4	Terminal residue
	Shibei Town, Zhongxiang City, Hubei Province	Hubei	Bendiwang	Terminal residue
	Huaxi District, Guiyang City, Guizhou Province	Guizhou	Zhongnong8	Residual dissipation and terminal residue
cabbage	Weiyang District, Xian City, Shanxi Province	Shanxi	Zhonggan22	Terminal residue
	Baishan Town, Changing District, Beijing	Beijing	Zhonggan21	Residual dissipation and terminal residue

Crop	Test address	Abbreviation of test sites	Crop varieties	Test types
cabbage	Taishan District, Tai'An City, Shandong Province	Shandong	Shenglv168	Terminal residue
	Zhongmou City, Zhengzhou City, Henan Province	Henan	Yafeilili	Terminal residue
	Luyang District. Hefei City, Anhui Province	Anhui	Dongfeng	Terminal residue
	Xinbang Town, Songjiang District, Shanghai Province	Shanghai	Chunfeng508	Residual dissipation and terminal residue
	Chunhua Town, Changsha City, Hunan Province	Hunan	Zhonggan19	Residual dissipation and terminal residue
	Duchang City, Jiujiang City, Jiangxi Province	Jiangxi	Xialian	Terminal residue
	Xixiangtang District, Nanning City, Guangxi Province	Guangxi	Bianqiu	Terminal residue
	Longlin City, Baise City, Guangxi Province	Chongqing	Zhonggan21	Terminal residue
	Huaxi District, Guizhou City, Guizhou Province	Guizhou	Qiangan1	Terminal residue
	Gaoming District, Foshan City, Guangdong Province	Guangdong	Zhonggan21	Residual dissipation and terminal residue
Cotton	Quzhou City, Handan City, Hebei Province	Hebei	Huamian5	Residual dissipation and terminal residue
	Daiyue District, Tai'An City, Shandong Province	Shandong	Lanhan	Residual dissipation and terminal residue
	Pingyuan District, Xinxiang City, Henan Province	Henan	Tianshanshengxue	Residual dissipation and terminal residue
	Chaohu City, Hefei City, Anhui Province	Anhui	Quanyinmian3	Terminal residue
	Yong'an Town, Liuyang City, Hunan Province	Hunan	Xipu818	Terminal residue
	Duchang City, Jiujiang City, Jiangxi Province	Jiangxi	Eza10	Terminal residue
	Shangzhuang Town, Haidian District, Beijing	Beijing	Huamian5	Terminal residue
	Shibei Town, Zhongxiang City, Hubei Province	Hubei	Tianshanshengxue	Residual dissipation and terminal residue
